# Sir George Newman on the School Child

**Published:** 1916-09-30

**Authors:** 


### SIR GEORGE NEWMAN ON THE SCHOOL CHILD.

### The Arousing of the National Conscience.

Seldom, to judge by the comments of the more responsible organs of the press, has the Annual Report of the Chief Medical Officer to the Board of Education aroused more general public interest and appreciation than that which has lately been presented to the House of Commons and is now published. To a considerable extent this is ascribable to the intrinsic excellence of the report, and to its brief and pithy introduction especially. But this is not, probably, by any means the whole of the explanation. The quickened conscience of the nation concerning its responsibilities towards the next generation is also in some large degree responsible; and whether this sensitiveness be in part due to the sense of realities hammered home by the appalling sacrifices of war, as seems likely, it is certainly also in part due to the spade-work during the past eight years of Sir George himself and of his subordinates and associates, paid and voluntary both included.

Sir George's reports are always worth reading, and in the ten paragraphs which form the introduction to this year's Blue-book he gives a most succinct account of present tendencies and future probabilities in the development of his department. If at times he emphasises the obvious, or is attracted by the facile phrase, at least he constantly stimulates the attention, and keeps principles, rather than details, firmly in view. And if he is to be judged solely by his effect upon the lay press (a misleading criterion admittedly) it will be allowed that this report is the most important of the series. This deep impression made upon ephemeral literature may, it is to be hoped, be accepted as an indication of a corresponding impression upon the public conscience, both individual and corporate; and if that hope is realised it should be speedily reflected in extra efficiency and extra attention of local education authorities in co-operation with their school medical officers and school teachers.

### Criticisms from the Schools.

It would not be quite accurate to give the impression that there are no dissentient voices to be heard. A headmaster, for instance, accuses Sir George (in the *Daily News*) of "the hugest blunder possible" in respect of certain statistics. Inasmuch, however, as the headmaster seriously misquotes Sir George, and clearly misapprehends the whole purport of the statement on which he bases his attack, it would be foolish to attach much importance to his objection. It is this critic, not the Chief Medical Officer of the Board, who has made a "ridiculous statement." Another school manager, writing to the *Times*, draws attention to two points arising out of the report, one of which is scarcely within the purview of the Board of Education at all (the destruction of verminous houses), and the other is probably not so overlooked as he inclines to suspect. Nor is the voice of criticism silent in Scotland.

It has been a catchword of party politics that London lags behind some of the provincial cities, and Birmingham especially has been quoted as an example of foresight in policy and munificence in equipment. This complaint against London is unjustified in essentials, even if in occasional details it has some substance; and it finds little encouragement in the pages of this report. Special difficulties exist in London, and although they are not all surmounted, there is every reason to believe that London's alleged lagging is much less real than interested politicians sometimes choose to admit. To take an instance at random, of interest for comparison with the position at Shrewsbury, it is stated that ringworm appears to be gradually diminishing in the Metropolis. The number of cases reported in each of the last five years has declined steadily from 6,215 in the first year of the series to 3,747 in the last. During the same time the proportion of cases treated by $x$-rays has risen from 30 to 50 per cent.; and Sir George commits himself definitely to the statement that this treatment is both the most effective and many times the most rapid. There are still many parents, it seems, who object on the score of possible brain injury; doubtless the most strenuous and inconvertible objectors are those whose ignorance is the most abysmal.

### The Verminous Child.

With regard to the detailed sources of invalidism or of physical ill-being amongst school children, the sections dealing with uncleanliness (which means infestation with vermin, not mere dirt) may be commended to the attention of all careful citizens. They deal with a repulsive topic, and reveal nothing that all connected with school children and educational problems among the working classes do not know already. But precisely because the subject is unsavoury and familiar it is apt to be glossed over and to escape consideration. To anyone who will study the horrible state of parental ignorance, neglect, and apathy set forth by Sir George Newman's tables, it will be at once apparent why the educational system of America, where all classes rub shoulders together in the public elementary schools, is out of the question in this country.

The expansion of Sir George Newman's staff during the past eight years has been very great, and would have been greater but for the all-absorbing war. At present it consists of 855 school medical officers and assistant medical officers. Besides these there are 144 ophthalmic surgeons, 35 aural surgeons, 217 dental surgeons, 29 $x$-ray specialists, and 20 anæsthetists. These figures exclude the 215 medical officers who form the staffs of the treatment centres in London; 103 of the 1,300 are women doctors, 88 of whom are whole-time officials. Besides the doctors 1,484 school nurses are employed, of whom 996 do whole-time service.

